# Ivermectin Prophylaxis Used for COVID-19: A Citywide, Prospective, Observational Study of 223,128 Subjects Using Propensity Score Matching

**DOI:** 10.7759/cureus.21272

**Published:** 2022-01-15

**Authors:** Lucy Kerr, Flavio A Cadegiani, Fernando Baldi, Raysildo B Lobo, Washington Luiz O Assagra, Fernando Carlos Proença, Pierre Kory, Jennifer A Hibberd, Juan J Chamie-Quintero

**Affiliations:** 1 Medicine, Instituto Kerr, São Paulo, BRA; 2 Clinical Endocrinology, Corpometria Institute, Brasilia, BRA; 3 Clinical Endocrinology, Applied Biology Inc, Irvine, USA; 4 Animal Sciences, Universidade Estadual de São Paulo (UNESP), São Paulo, BRA; 5 Genetics, Universidade de São Paulo, Ribeirão Preto, BRA; 6 Genetics, Centro Técnico de Avaliação Genômica - C.T.A.G., Ribeirão Preto, BRA; 7 Bioinformatics, Itajaí City Hall, Itajaí, BRA; 8 Internal Medicine, Front Line COVID-19 Critical Care Alliance (FLCCC), Madison, USA; 9 Dentistry, University of Toronto, Toronto, CAN; 10 Data Analysis, Universidad EAFIT, Medellín, COL

**Keywords:** coronavirus, prevention, prophylaxis, ivermectin, sars-cov-2, covid-19

## Abstract

Background: Ivermectin has demonstrated different mechanisms of action that potentially protect from both coronavirus disease 2019 (COVID-19) infection and COVID-19-related comorbidities. Based on the studies suggesting efficacy in prophylaxis combined with the known safety profile of ivermectin, a citywide prevention program using ivermectin for COVID-19 was implemented in Itajaí, a southern city in Brazil in the state of Santa Catarina. The objective of this study was to evaluate the impact of regular ivermectin use on subsequent COVID-19 infection and mortality rates.

Materials and methods: We analyzed data from a prospective, observational study of the citywide COVID-19 prevention with ivermectin program, which was conducted between July 2020 and December 2020 in Itajaí, Brazil. Study design, institutional review board approval, and analysis of registry data occurred after completion of the program. The program consisted of inviting the entire population of Itajaí to a medical visit to enroll in the program and to compile baseline, personal, demographic, and medical information. In the absence of contraindications, ivermectin was offered as an optional treatment to be taken for two consecutive days every 15 days at a dose of 0.2 mg/kg/day. In cases where a participating citizen of Itajaí became ill with COVID-19, they were recommended not to use ivermectin or any other medication in early outpatient treatment. Clinical outcomes of infection, hospitalization, and death were automatically reported and entered into the registry in real time. Study analysis consisted of comparing ivermectin users with non-users using cohorts of infected patients propensity score-matched by age, sex, and comorbidities. COVID-19 infection and mortality rates were analyzed with and without the use of propensity score matching (PSM).

Results: Of the 223,128 citizens of Itajaí considered for the study, a total of 159,561 subjects were included in the analysis: 113,845 (71.3%) regular ivermectin users and 45,716 (23.3%) non-users. Of these, 4,311 ivermectin users were infected, among which 4,197 were from the city of Itajaí (3.7% infection rate), and 3,034 non-users (from Itajaí) were infected (6.6% infection rate), with a 44% reduction in COVID-19 infection rate (risk ratio [RR], 0.56; 95% confidence interval (95% CI), 0.53-0.58; p < 0.0001). Using PSM, two cohorts of 3,034 subjects suffering from COVID-19 infection were compared. The regular use of ivermectin led to a 68% reduction in COVID-19 mortality (25 [0.8%] versus 79 [2.6%] among ivermectin non-users; RR, 0.32; 95% CI, 0.20-0.49; p < 0.0001). When adjusted for residual variables, reduction in mortality rate was 70% (RR, 0.30; 95% CI, 0.19-0.46; p < 0.0001). There was a 56% reduction in hospitalization rate (44 versus 99 hospitalizations among ivermectin users and non-users, respectively; RR, 0.44; 95% CI, 0.31-0.63; p < 0.0001). After adjustment for residual variables, reduction in hospitalization rate was 67% (RR, 0.33; 95% CI, 023-0.66; p < 0.0001).

Conclusion: In this large PSM study, regular use of ivermectin as a prophylactic agent was associated with significantly reduced COVID-19 infection, hospitalization, and mortality rates.

## Introduction

Ivermectin has been demonstrated to have not only extensive anti-parasitic actions [1,2], but also anti-viral, anti-bacterial, and anti-protozoan properties. Ivermectin has been long proposed for use as a repurposed antiviral agent [3-6]. Indeed, antiviral effects of ivermectin have been reported against both RNA and DNA types of viruses, including HIV-1, yellow fever, Japanese encephalitis, tick-borne encephalitis, West Nile, Zika, dengue fever, chikungunya, Venezuelan equine encephalitis, and the pseudorabies virus [3,5,7,8], as well as functioning in regulation of proteins involved in antiviral responses [8].

Additional actions of ivermectin described include agonism activity to the liver X receptor (LXR) and farnesoid X receptor (FXR), with multiple potential metabolic benefits [9,10]; neuronal regeneration [11,12], prevention of muscle hypoxia [13], and actions on specific sites, including interferon (INF) [14], nuclear factor-κB (NF-κB), lipopolysaccharide (LPS) [15], and Janus kinase/signal transducer and activator of transcription (JAK-STAT) and PAI-1 pathway [16,17]; generation of P21 activated kinase 1 (PAK-1) [18,19]; reduction of interleukin-6 (IL-6) levels [15]; allosteric modulation of P2X4 receptor [20]; inhibition of high mobility group box 1 (HMGB1) [21,22]; and suppression of mucus hypersecretion, diminished recruitment of immune cells, and production of cytokines in the lung [23]. Ivermectin is also described to induce T helper 1 cell (Th1)-type immune response against protozoan infections [24], and anti-coagulant action through binding to the S protein of some viruses [25].

The hypothesis that ivermectin could be protective against coronavirus disease 2019 (COVID-19) is substantiated by its multi-pathway, anti-inflammatory effects [15,26], and multi-antiviral mechanisms. COVID-19 pathogenesis is largely understood as an inflammation-mediated hemagglutinating infection disrupting pulmonary, vascular, and endothelial systems, leading to a multi-systemic disease. In vitro and in silico, ivermectin has demonstrated anti-severe acute respiratory syndrome coronavirus 2 activity through more than 20 direct and indirect mechanisms [2,27,28].

Ivermectin has demonstrated preliminary protective effects against severe acute respiratory syndrome coronavirus 2 (SARS-CoV-2) infection in terms of reducing times to clinical recovery and rates of disease progression and mortality [2,29,30]. However, more robust studies with larger sample sizes are still recommended to confirm the possible beneficial effects of ivermectin in COVID-19.

Since the onset of the COVID-19 pandemic, the use of inexpensive options based on a consistently beneficial signal of efficacy, a well-established safety profile, and favorable cost-effectiveness, ivermectin is a highly attractive intervention for the patient-centered medicine practiced by frontline clinicians, with use aligning strongly with the bioethical principles for medical practice outlined in Article 36 of the Declaration of Helsinki [31].

However, despite this favorable risk/benefit profile and absence of therapeutic alternatives, ivermectin is yet to be approved for prophylaxis and treatment of COVID-19 by agencies throughout the world, including FDA (USA), European Medicines Agency (EMA; Europe), and ANVISA (Agência Nacional de Vigilância Sanitária - Brazilian Health Regulatory Agency; Brazil).

The ability to prescribe ivermectin or any other off-label drug for COVID-19 has long been at the discretion of frontline physicians once all risks, uncertainties, potential benefits, and patients’ rights are exposed, and informed consent has been obtained. Of particular note, in Brazil, this follows the medical autonomy to determine the best therapeutic strategies for individuals, as per the Medical Code of Ethics of the Brazilian Board of Medical Doctors, the Federal Council of Medicine - Conselho Federal de Medicina (CFM), that determines the obligations and rights of medical doctors in Brazil [32].

Since vaccines for COVID-19 were not available in Brazil until 2021, and because of the lack of prophylactic alternatives in the absence of vaccines, Itajaí, a city in the southern Brazilian state of Santa Catarina, initiated a population-wide government program for COVID-19 prophylaxis. The medical-focused decision parameters established are based on the distribution of ivermectin to whole populations in different countries. To ensure the safety of the population, a well-controlled computer program was developed to compile and maintain all relevant demographic and clinical data (detailed in the Materials & Methods section). The use of ivermectin was optional and based on patients’ preferences, given its benefits as a preventative agent was unproven.

This study’s objective is to assess the impact on important clinical outcomes when ivermectin is used as prophylaxis for COVID-19. The prophylaxis program occurred in addition to the standard non-pharmacological strategies of masking and social distancing, as part of a citywide program conducted in outpatient settings.

## Materials and methods

Study population

This was a prospective, observational study. Although study design, institutional review board (IRB) approval, and data analysis occurred after completion of the voluntary prophylaxis program, all data were collected prospectively in real time with mandated reporting to the registry of all events as they occurred during the citywide governmental COVID-19 prevention with ivermectin program, from July 2020 to December 2020, developed in the city of Itajaí, in the state of Santa Catarina, Brazil. Demographic and clinical data were reported from medical records of patients followed in a large outpatient setting (a provisional outpatient clinic set in the Convention Center of Itajaí) and several secondary outpatient settings, as part of the universal health system (Sistema Único de Saúde [SUS]).

The objective was to determine the number of patients affected by COVID-19 (positivity rate of reverse transcription-polymerase chain reaction [RT-PCR] for SARS-CoV-2), risk of death due to COVID-19 (whether infected or not), and COVID-19 mortality rate (risk of death from COVID-19) of those who used and did not use ivermectin prophylactically for COVID-19. These data were stratified by age, sex, presence of comorbidities, and correlated demographic characteristics.

The present retrospective analysis of the prospectively collected data was approved by the National Research Ethics Council (CONEP) under the number 4.821.082 with the project number CAAE: 47124221.2.0000.5485. Although study design, IRB approval, and data analysis occurred after completion of the voluntary prophylaxis program, all data were collected prospectively in real-time with mandated reporting to the registry of all events as they occurred during the citywide governmental COVID-19 prevention with ivermectin program, from July 7, 2020, to December 2, 2020, developed in the city of Itajaí, in the state of Santa Catarina, Brazil.

Study procedures and data collection

Optional, voluntary prophylactic use of ivermectin was offered to patients during regular medical visits between July 7, 2020, and December 2, 2020, in 35 different sites, including 34 local SUS health centers and a large temporary patient setting 24/7. Doctors working in these sites were free to prescribe ivermectin prophylactically. Subjects that did not use ivermectin either refused or their primary care physicians opted not to offer ivermectin.

To avoid underreported data, strict procedure sequencing was followed: (1) registration and recording of patient data, documented by assistants; (2) weighing subjects (subject's weight was essential to calculate the appropriate dose of ivermectin); (3) brief medical evaluation of past medical history, comorbidities, use of medications, and contraindications to drugs; and (4) medical prescription with prophylactic doses of ivermectin (within recommended usual, safe doses of ivermectin), according to medical judgment and following a subject’s informed consent related to potential benefits, risks, and side effects. All details of this citywide program and campaign had been previously agreed upon between the city local department of the National Healthcare System (SUS), city mayor, and local public prosecutors.

Regarding drug interactions with ivermectin, the use of warfarin was a contraindication for prophylaxis with ivermectin due to drug interactions. Subjects under chronic use of glucocorticoids, protease inhibitors, and anti-epileptics were recommended to schedule regular medical visits every six to eight weeks. Subjects were recommended to inform medical doctors about the use of ivermectin, in case one or more of the following medications were prescribed: warfarin, azithromycin, dexamethasone, prednisone, or prednisolone (hydrocortisone or cortisone are not commercially available in regular pharmacies in Brazil).

The following variables were analyzed: (1) age, (2) sex, (3) previous diseases (myocardial infarction [MI] and stroke), (4) pre-existing comorbidities (type 2 diabetes [T2D], asthma, chronic obstructive pulmonary disease [COPD], hypertension, dyslipidemia, cardiovascular diseases [CVD], cancer [any type], and other pulmonary diseases), and (5) smoking. Variables were adjusted as confounding factors and used as variables for balancing and matching groups for propensity score matching (PSM).

Patients who presented signs or the diagnosis of COVID-19 before July 7, 2020, were excluded from the sample. Other exclusion criteria were contraindications to ivermectin and subjects below 18 years of age. The dose and frequency of ivermectin treatment was 0.2 mg/kg/day; i.e., giving one 6 mg tablet for every 30 kg for two consecutive days every 15 days.

During the study, subjects who were diagnosed with COVID-19 underwent a specific medical visit to assess COVID-19 clinical manifestations and severity. All subjects were recommended not to use ivermectin, nitazoxanide, hydroxychloroquine, spironolactone, or any other drug claimed to be effective against COVID-19. The city did not provide or support any specific pharmacological outpatient treatment for subjects infected with COVID-19.

They were questioned for the presence of common COVID-19 symptoms. These included chills, high-grade fever, cough, myalgia, fatigue, anosmia, ageusia, sore throat, headache, nasal congestion, sneeze, runny nose, hemoptysis, nausea, vomiting, abdominal pain, diarrhea, cutaneous rash, arthralgia, chest pain, eye pain and pinkeye, and presence of alert signs, including shortness of breath, signs of hypoxia, signs of coagulation abnormalities, and an altered level of consciousness. Systolic and diastolic blood pressure, heart rate, respiratory rate, oxygen saturation, and axillary temperature were measured. The same signs and symptoms and vital signs were collected at each following medical visit during COVID-19. Individual data were compiled and reviewed by the researchers.

Registry data of all patient records from the city of Itajaí between July 7, 2020, and December 2, 2020, including those who used ivermectin and did not use ivermectin were reviewed. All subjects who tested positive for COVID-19 in the city of Itajaí during the study were considered for this analysis. Of the infected subjects, two groups were considered: subjects who used ivermectin prophylactically (treated group) and subjects who did not use ivermectin prophylactically (untreated group). Missing data from patients were clarified with patients or relatives directly, via phone or in person, by the investigators. Since this is a citywide program, all recorded data must have matched the exact number of COVID-19 cases and deaths of the city. This strict interval avoids differences in terms of periods of exposure.

Due to the uncertainty of reinfection with COVID-19, subjects with a history of previous COVID-19 did not participate in the program although they were still permitted to use ivermectin prophylactically. Limiting parameters of the government system allowed the recording of a first episode of COVID-19 infection only. Subjects below 18 years old and subjects with a diagnosis of COVID-19 before July 7, 2020, were excluded from all datasets and analyses.

From the registry of the city population (223,128 inhabitants), subjects below 18 years old (61,583 subjects) were removed. Of the 161,545 subjects above 18 years old from the city of Itajaí, we removed the 1,984 COVID-19 cases that occurred before July 7, 2020, and 159,561 subjects remained. Subjects above 18 years old were considered those who were born before June 30, 2002.

A total of 147,223 subjects participated in the program of ivermectin prophylaxis used for COVID-19. Of these, 24,304 subjects were below 18 years old. Of the 122,919 ivermectin users above 18 years old, 8,346 were from other cities, and 728 had COVID-19 before July 7, 2020, although they used ivermectin afterward. In total, 113,845 subjects that participated in the program remained in the dataset. The 45,716 non-participants, remaining subjects among the 159,561 subjects, were considered as the ivermectin non-users.

Finally, citywide COVID-19 hospitalization and mortality rates of Itajaí were compared between the period before the program (before July 7, 2020) and during the program (between July 7, 2020, and December 2, 2020) aiming to evaluate whether a program of prophylaxis with ivermectin for COVID-19 would cause a positive impact in the overall numbers of the city, despite only partial adoption. Chances of dying of COVID-19 in the overall population, according to use or non-use of ivermectin (irrespective of COVID-19 infection) were only calculated prior to matching. Conversely, the mortality rate among those who were infected by the SARS-CoV-2 was calculated for both pre and post-matched cohorts.

Hospitalization and mortality rates before matching groups, the mortality rate in subpopulations before and after PSM, and the Strengthening the Reporting of Observational Studies in Epidemiology (STROBE) checklist are presented in the Appendix.

Statistical analysis

The full underlying data for the present analysis were analyzed by two independent statisticians, and discrepancies were evaluated by a third statistics expert. In this outpatient study of those who tested positive for SARS-CoV-2, the mortality rate was evaluated according to each parameter that was adjusted against other variables (for multivariate regression analysis) and used for balancing and matching groups, including age intervals, sex, history of smoking, prophylactic ivermectin use, T2D, asthma, COPD, cardiovascular diseases and other pulmonary diseases, hypertension, current cancer (any type), and history of stroke and/or MI.

Before matching, a generalized linear mixed model was employed, assuming the binomial distribution for the residues and including the fixed classificatory effects of each of these parameters. Age intervals were adjusted for the evaluation of ivermectin prophylactic use as an independent predictor of death from COVID-19. Unadjusted and multivariate Poisson-adjusted probabilities to survive from COVID-19 (p-value), according to each parameter, were provided.

PSM was performed for mortality risk between ivermectin and non-ivermectin users. COVID-19 infection rate and risk of dying were also calculated for variables. After PSM, a second adjustment ("double adjustment") with multivariate linear regression was performed for residual variables [33,34].

There were no missing data since the registry system design mandated that all data variables be filled to be formally included in the registry. Only erroneously entered (illogical) data were found. In such instances, a medical record review was performed to obtain accurate data. The program used for the analysis was the Statistical Analysis Software (SAS/STAT) (SAS Institute Inc., Cary, NC). For transparency reasons, two datasets of the 7,345 COVID-19 cases and the 113,845 participating subjects considered for the present analysis will be made public upon peer-reviewed publication.

## Results

**Figure 1:**
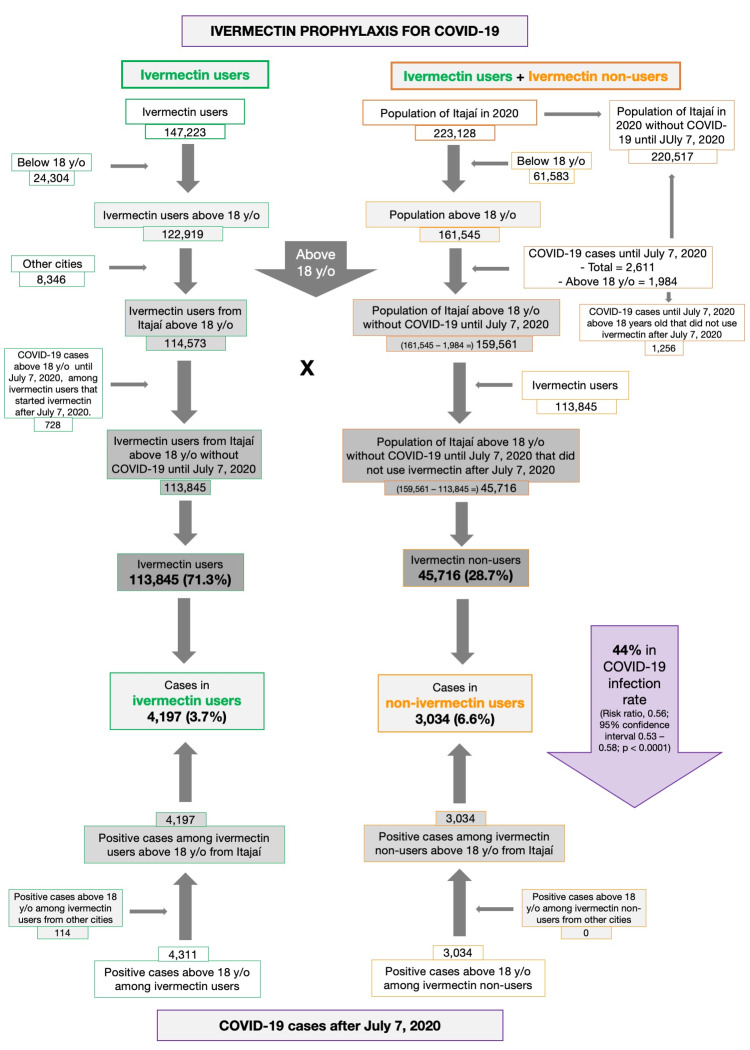
Underlying data for the study on ivermectin prophylaxis used for COVID-19.

A detailed description of the data considered for the present analysis is illustrated in Figure 1. Of the 220,517 citizens of Itajaí without COVID-19 until July 7, 2020, 159,561 were above 18 years old. Of the 159,561 citizens above 18 years old without COVID-19 until July 7, 2020, 113,845 (71.3% of the population above 18 years old) received ivermectin before being infected by COVID-19. A total of 45,716 citizens (28.7%) did not receive or did not want to receive ivermectin during the program, including as a prophylactic or as a treatment after having COVID-19.

Of the 113,845 prophylaxed subjects from the city of Itajaí, 4,197 had a positive RT-PCR SARS-CoV-2 (3.7% infection rate), while 3,034 of the 37,027 untreated subjects had positive RT-PCR SARS-CoV-2 (6.6% infection rate), a 44% reduction in COVID-19 infection rate (risk ratio [RR], 0.56; 95% confidence interval (95% CI), 0.53-0.58; p < 0.0001). An addition of 114 subjects who used ivermectin and were infected were originally from other cities but were registered as part of the program, in a total of 4,311 positive cases among ivermectin users. For the present analysis, the 4,311 positive cases among subjects that used ivermectin and 3,034 cases among subjects that did not use ivermectin were considered. After PSM, two cohorts of 3,034 subjects were created.

**Figure 2:**
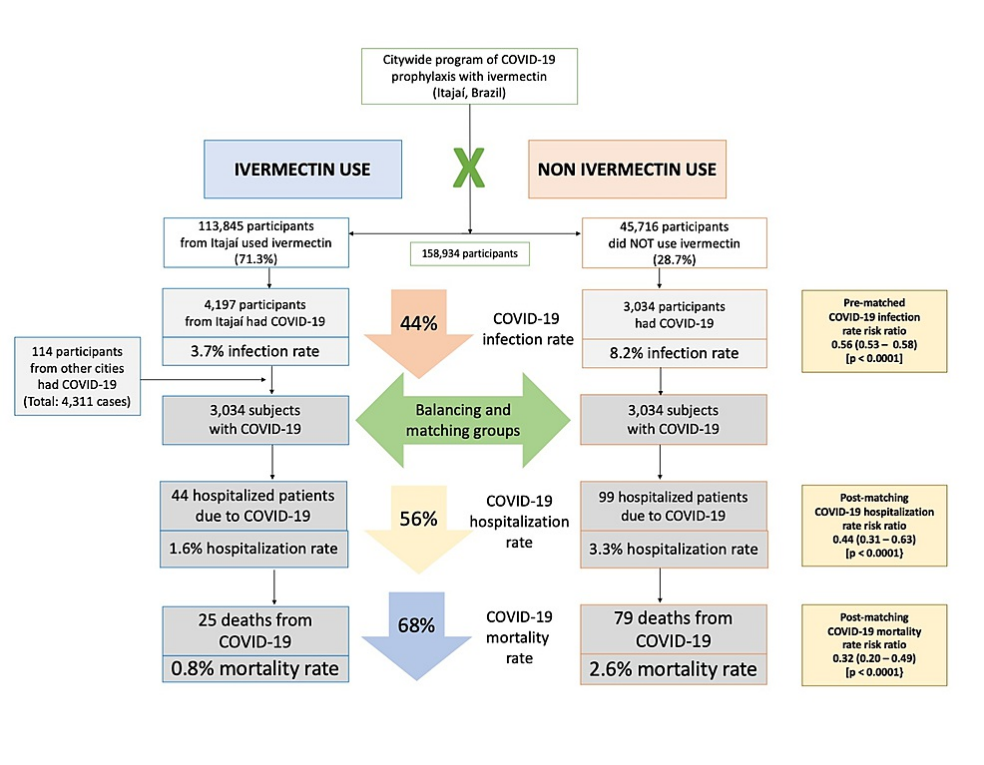
Summary of the findings.

Baseline characteristics of the 7,345 subjects included prior to PSM and the baseline characteristics of the 6,068 subjects in the matched groups are shown in Table 1. Prior to PSM, ivermectin users had a higher percentage of subjects over 50 years old (p < 0.0001), higher prevalence of T2D (p = 0.0004), hypertension (p < 0.0001), and CVD (p = 0.03), and a higher percentage of Caucasians (p = 0.004), than non-users. After PSM, all baseline parameters were similar between groups. Figure 2 summarizes the main findings of this study.

**Table 1:** Baseline characteristics of subjects enrolled in the study before matching and after propensity score matching. COPD = chronic obstructive pulmonary disease; CVD = cardiovascular disease; MI = myocardial infarction; SD = standard deviation.

	Pre-matching				Propensity score-matched		
	Overall (n = 7,345)	Ivermectin users (n = 4,311)	Non-ivermectin users (n = 3,034)	p-value	Overall (n = 6,068)	Ivermectin users (n = 3,034)	Non-ivermectin users (n = 3,034)
Age							
Mean ± SD	42.0 ± 14.7	43.5 ± 14.9	39.8 ± 14.2	<0.0001	39.7 ± 14.0	3967 ± 13.8	39.8 ± 14.2
<30 years old	1,730 (23.6%)	886 (20.5%)	844 (27.8%)		1,691 (27.9%)	844 (27.9%)	847 (27.8%)
30-50 years old	3,703 (50.4%)	2,121 (49.2%)	1,582 (52.2%)		3,155 (52.0%)	1,573 (51.9%)	1,582 (52.1%)
>50 years old	1,912 (26.0%)	1,304 (30.3%)	608 (20.0%)		1,222 (20,1%)	614 (20.2%)	608 (20.1%)
Sex				0.31			
Female	3,983 (54.2%)	2,359 (54.7%)	1,624 (53.5%)		3,231 (53.2%)	1,607 (53.0%)	1,624 (53.5%)
Male	3,362 (45.8%)	1,952 (45.3%)	1,410 (46.5%)		2,837 (46.8%)	1,427 (47.0%)	1,410 (46.5%)
Race							
Caucasians	5,437 (74.0%)	3,245 (75.3%)	2,192 (72.2%)	0.004	4,398 (72.5%)	2,206 (72.7%)	2,192 (72.3%)
Afro-Brazilians	209 (2.8%)	109 (2.5%)	100 (3.3%)	0.052	193 (3.2%)	93 (3.1%)	100 (3.3%)
Mixed	1,583 (22.6%)	901 (20.9%)	682 (22.5%)	0.10	1,364 (22.5%)	93 (3.1%)	100 (3.3%)
Asian-Brazilians	116 (1.6%)	56 (1.3%)	60 (2.0%)	0.023	113 (1.9%)	53 (1.8%)	60 (2.0%)
Type 2 diabetes				0.0004			
Yes	214 (2.9%)	151 (3.5%)	63 (2.1%)		141 (2.3%)	78 (2.6%)	63 (2.1%)
No	7,131 (97.1%)	4,160 (96.5%)	2,971 (97.9%)		5,927 (97.7%)	2,956 (97.4%)	2,971 (97.9%)
Asthma				0.067			
Yes	26 (0.3%)	20 (0.5%)	6 (0.2%)		21 (0.3%)	15 (0.5%)	6 (0.2%)
No	7,319 (99.7%)	4,291 (99.5%)	3,028 (99.8%)		6,047 (99.7%)	3,019 (99.5%)	3,028 (99.8%)
COPD				0.72			
Yes	13 (0.2%)	7 (0.2%)	6 (0.2%)		12 (0.2%)	6 (0.2%)	6 (0.2%)
No	7,332 (99.8%)	4,304 (99.8%)	3,028 (99.8%)		6,056 (99.8%)	3,028 (99.8%)	3,028 (99.8%)
Hypertension				<0.0001			
Yes	528 (7.2%)	362 (8.4%)	166 (5.5%)		343 (5.6%)	177 (5.8%)	166 (5.5%)
No	6,817 (92.8%)	3,949 (91.6%)	2,868 (94.5%)		5,725 (94.4%)	2,857 (94.2%)	2,868 (94.5%)
CVD				0.03			
Yes	56 (0.8%)	41 (1.0%)	15 (0.5%)		32 (0.5%)	17 (0.6%)	15 (0.5%)
No	7,289 (99.2%)	4,270 (99.0%)	3,019 (99.5%)		6,036 (99.5%)	3,017 (99.4%)	3,019 (99.5%)
Other pulmonary diseases				0.53			
Yes	15 (0.2%)	10 (0.2%)	5 (0.2%)		9 (0.1%)	4 (0.1%)	5 (0.1%)
No	7,330 (99.8%)	4,301 (99.8%)	3,029 (99.8%)		6,059 (99.9%)	3,030 (99.9%)	3,029 (99.9%)
Cancer (any type)				0.66			
Yes	32 (0.4%)	20 (0.5%)	12 (0.4%)		22 (0.4%)	10 (0.3%)	12 (0.4%)
No	7,313 (99.6%)	4,291 (99.5%)	3,023 (99.6%)		6,046 (99.6%)	3,024 (99.7%)	3,022 (99.6%)
Current smoking				0.76			
Yes	110 (1.5%)	63 (1.5%)	47 (1.5%)		95 (1.6%)	48 (1.6%)	47 (1.6%)
No	7,235 (98.5%)	4,248 (98.5%)	2,987 (98.5%)		5,973 (98.4%)	2,986 (98.4%)	2,987 (98.4%)
History of MI				0.26			
Yes	15 (0.2%)	11 (0.3%)	4 (0.1%)		8 (0.1%)	4 (0.1%)	4 (0.1%)
No	7,330 (99.8%)	4,300 (99.7%)	3,030 (99.9%)		6,060 (99.9%)	3,030 (99.9%)	3,030 (99.9%)
History of stroke				0.56			
Yes	21 (0.3%)	11 (0.3%)	10 (0.3%)		21 (0.4%)	11 (0.4%)	10 (0.3%)
No	7,324 (99.7%)	4,300 (99.7%)	3,024 (99.7%)		6,047 (99.6%)	3,023 (99.6%)	3,024 (99.7%)

Hospitalization and mortality rates in ivermectin users and non-users in propensity score-matched analysis

As described in Table 2, after employing PSM, of the 6,068 subjects (3,034 in each group), there were 44 hospitalizations among ivermectin users (1.6% hospitalization rate) and 99 hospitalizations (3.3% hospitalization rate) among ivermectin non-users, a 56% reduction in hospitalization rate (RR, 0.44; 95% CI, 0.31-0.63). When adjustment for variables was employed, the reduction in hospitalization rate was 67% (RR, 0.33; 95% CI, 023-0.66; p < 0.0001).

**Table 2:** Propensity score-matched hospitalization and mortality rate among ivermectin users and non-users. IVM = ivermectin; PSM = propensity score matching. * Only subjects hospitalized in public hospitals. ** All deaths, including from public and private hospitals, and in-home.

		Overall	IVM users	Non-IVM users	PSM mortality risk ratio (95% CI) and p-value [p]	Adjusted PSM mortality risk ratio (95% CI) and p-value [p]
COVID-19 infection	Infected population (n)	6,068	3,034	3,034	-	-
COVID-19 hospitalization	Hospitalization due to COVID-19	143	44	99	-	-
	Hospitalization rate* (in case of COVID-19) (%)	2.3%	1.6%	3.3%	0.44 (0.31-0.63) [<0.0001]	0.33 (0.23-0.46) [<0.0001]
COVID-19 death	COVID-19 deaths (n)**	104	25	79	-	-
	Mortality rate (among infected subjects) (%)	1.7%	0.8%	2.6%	0.32 (0.20-0.49) [<0.0001]	0.30 (0.19-0.46) [<0.0001]

There were 25 deaths among ivermectin users (0.8% mortality rate) and 79 deaths among non-ivermectin users (2.6% mortality rate), a 68% reduction in mortality rate (RR, 0.32; 95% CI, 0.20-0.49). When PSM was adjusted, reduction in mortality rate was 70% (RR, 0.30; 95% CI, 0.19-0.46; p < 0.0001).

Determinants of COVID-19 mortality through propensity score-matched analysis

Table 3 describes the resulting risk factors for COVID-19 death amongst the overall population through PSM analysis. Risk factors for mortality in COVID-19 included aging (p < 0.0001), male sex (p = 0.015), T2D (p < 0.0001), hypertension (p < 0.0001), asthma (p = 0.011), COPD (p < 0.0001), other pulmonary diseases (p = 0.048), history of MI (p = 0.034), and history of stroke (p < 0.0001). To detect independent risk factors, post-PSM adjustment for variables showed that ivermectin (p < 0.0001; 70% reduction in mortality risk) and female sex (p = 0.022; 38% reduction in mortality risk) were protectors, whereas T2D (p = 0.041; 79% increase in mortality risk), hypertension (p = 0.008; 98% increase in mortality risk), and, marginally, other pulmonary diseases (p = 0.061; 468% increase in mortality risk) and history of stroke (p = 0.054; 97% increase in mortality risk) were identified as independent risk factors.

**Table 3:** Propensity score-matched COVID-19 mortality rate according to each characteristic in the overall population, ivermectin users, and non-users. COPD = chronic obstructive pulmonary disease; CVD = cardiovascular disease; MI = myocardial infarction.

	Propensity score-matched groups			
Variable	Overall (n = 6,068)	Death (%)	Unadjusted COVID-19 mortality risk ratio and p-value [p]	Multivariate adjusted COVID-19 mortality risk ratio and p-value [p]
Ivermectin use - n (%)			0.32 (0.20-0.49) [<0.0001]	0.30 (0.19-0.46) [<0.0001]
Yes	3,034	25 (0.8%)		
No	3,034	79 (2.6%)		
Age - n (%)			[<0.0001]	[<0.0001]
<30 years old	1,691	1 (0.1%)		
30-50 years old	3,155	12 (0.4%)		
>50 years old	1,222	91 (7.4%)		
Sex - n (%)			0.62 (0.42-0.91) [0.015]	0.64 (0.44-0.93) [0.022]
Female	3,231	43 (1.3%)		
Male	2,837	61 (2.2%)		
Race - n (%)			[0.24]	[0.44]
Caucasians	4,398	79 (1.8%)		
Afro-Brazilians	193	6 (3.1%)		
Mixed	1.364	17 (1.3%)		
Asian-Brazilians	113	2 (1.9%)		
Type 2 diabetes - n (%)			10.0 (6.32-15.8) [<0.0001]	1.79 (1.03-3.12) [0.041]
Yes	141	20 (14.2%)		
No	5,927	84 (1.4%)		
Hypertension - n (%)			8.83 (5.99-13.0) [< 0.0001]	1.98 (1.19-3.30) [0.008]
Yes	343	36 (10.5%)		
No	5,725	68 (1.2%)		
Asthma - n (%)			5.64 (1.49-21.4) [0.011]	1.74 (0.52-5.81) [0.36]
Yes	21	2 (9.5%)		
No	6,047	102 (1.7%)		
COPD - n (%)			15.0 (5.52-40.7) [<0.0001]	1.71 (0.68-4.31) [0.25]
Yes	12	3 (25.0%)		
No	6,056	101 (1.7%)		
Cardiovascular diseases - n (%)			7.54 (2.96-19.3) [<0.0001]	1.22 (0.44-3.37) [0.70]
Yes	32	4 (12.5%)		
No	6,036	100 (1.7%)		
Other pulmonary diseases - n (%)			6.54 (1.02-41.9) [0.048]	5.68 (0.92-35.0) [0.061]
Yes	9	1 (11.1%)		
No	6,059	103 (1.7%)		
Cancer (any type) - n (%)			2.67 (0.39-18.3) [0.32]	1.97 (0.30-12.9) [0.48]
Yes	22	1 (4.6%)		
No	6,046	103 (1.7%)		
Current smoking - n (%)			1.23 (0.31-4.92) [0.77]	0.36 (0.08-1.70) [0.20]
Yes	95	2 (2.1%)		
No	5,973	102 (1.7%)		
History of MI - n (%)			7.35 (1.16-46.5) [0.034]	1.91 (0.17-21.6) [0.60]
Yes	8	1 (12.5%)		
No	6,060	103 (1.7%)		
History of stroke - n (%)			17.6 (8.72-35.7) [< 0.0001]	1.97 (0.99-3.92) [0.054]
Yes	21	6 (28.6%)		
No	6,047	98 (1.6%)		

In a comparison of citywide COVID-19 hospitalization rates prior to and during the program, COVID-19 mortality decreased from 6.8% before the program with prophylactic use of ivermectin, to 1.8% after its beginning (RR, 0.27; 95% CI, 0.21-0.33; p < 0.0001), and in COVID-19 mortality rate, from 3.4% to 1.4% (RR, 0.41; 95% CI, 0.31-0.55; p < 0.0001) (Table 4).

**Table 4:** Hospitalization and mortality rates registered in the city of Itajaí, Brazil, before versus after the beginning of the citywide program with ivermectin use as prophylaxis for COVID-19, independent of the ivermectin use status.

	Overall	Until July 30th	After July 30th	Relative risk ratio (95% CI)	p-value
Infected COVID-19 population (n)	9,956	2,663	7,293	-	-
Infected non-hospitalized COVID-19 population (n)	9,641	2,481	7,160	-	-
Hospitalized COVID-19 population (n)	315	182	133	-	-
COVID-19 hospitalization rate COVID-19 (%)	3.2%	6.8%	1.8%	0.27 (0.21-0.33)	<0.0001
Overall number of COVID-19 deaths	192	90	102	-	-
Overall mortality rate (%)	1.9%	3.4%	1.4%	0.41 (0.31-0.55)	<0.0001

## Discussion

This prospective, citywide COVID-19 ivermectin prophylaxis program resulted in significant reductions in COVID-19 infections, hospitalizations, and deaths. The ivermectin non-users were two times more likely to die of COVID-19 than ivermectin users in the overall population analysis. Since groups were compared for the exposure during the same period, in a parallel manner, changes in transmission rates would affect ivermectin users and non-users equally.

The city of Itajaí, in the state of Santa Catarina, Brazil, started a citywide program of prophylaxis with ivermectin in July 2020 as part of several initiatives to reduce the burden of COVID-19. The use of ivermectin was based on the existing literature at that time and on the virtual absence of risks. The National Health System (SUS) functions as full healthcare support to the entire population allowed the city to establish a non-restricted population program. This program included a support structure consisting of a large outpatient clinic located at the Convention Center of Itajaí. This outpatient clinic became the main locale of assistance for COVID-19 patients, supported by multiple public facilities where general practitioners regularly saw patients.

The use of ivermectin was optional unless contraindicated and given upon medical discretion. A structured medical-based program with a medical visit and evaluation of basic demographic characteristics and comorbidities offered ivermectin as optional prophylaxis to those who agreed to participate in this preventive treatment program. Health status was assessed and data were entered prospectively throughout the period of the program, in a fully digitized system provided by the National Health System (SUS). Since the system existed prior to the pandemic, a significant number of the population were already registered with their health information, including past and current diseases, use of medications, and other characteristics. The adaptations made to the SUS for the pandemic preparedness, prior to the initiation of this ivermectin outpatient program, allowed a structured, well-organized collection of the data that monitored any missing values, reinforcing the reliability of the results.

An important conservative bias was present. Major risk factors for severe COVID-19 and mortality due to COVID-19, including aging, diabetes, and hypertension, were more present among ivermectin users, which may have underestimated the benefits of ivermectin as it was demonstrated to be particularly effective in subjects above 49 years old in terms of reduction of absolute risk, which corresponds to the group at the highest risk for COVID-19. This allows the understanding that prophylactic use of ivermectin can be particularly impactful in older subjects. In addition, ivermectin seemed to reduce the exceeding risk of hypertension, T2D, and other diseases.

In accordance with the literature, subjects with higher age, diabetes, and males were less likely to survive (p < 0.05 for all), and only aging remained as an independent risk factor after PSM (p < 0.0001). However, prophylactic ivermectin use appears to mitigate the additional risk of COVID-19 death due to T2D, hypertension, and cardiovascular diseases.

The narrative that using preventive and early treatment therapies will have people relax their caution of remaining socially and physically distanced to allow more COVID-19-related infections is not supported here. These study data demonstrate that the use of preventive ivermectin significantly lowers the infection rate and that benefits outweigh the speculated increased risk of changes in social behaviors. Hence, we can speculate that the prophylactic use of ivermectin could play an important role in the reduction of the pandemic burden.

Even after adjustments to measure the most relevant variables that could influence COVID-19-related outcomes, including age, sex, comorbidities, and habits, aiming to avoid overestimation of the effects of ivermectin and to resemble a randomized clinical trial, prophylactic ivermectin proved to be protective for the overall population, with a reduction of 68% in mortality rate and p < 0.0001 after employment of PSM.

The protection provided by ivermectin when used prophylactically for COVID-19 may have reflected in the reduction in COVID-19 hospitalization and mortality rates observed at a population level. Compared to before the beginning of the program, COVID-19 hospitalization and mortality rates were reduced by 73% and 59%, respectively (p < 0.0001 for both). These reductions were obtained when the overall population and the number of COVID-19 cases, hospitalizations, and deaths in the city of Itajaí were considered, irrespective of the percentage of patients using ivermectin prophylactically. There were no changes in SARS-CoV-2 variants, infectivity, and pathogenicity before and during the program.

When compared to all other major cities in the state of Santa Catarina, differences in COVID-19 mortality rate before July 7, 2020, and between July 7, 2020, and December 21, 2020, Itajaí was ranked number one [35]. These results indicate that medical-based optional prescription and citywide covered ivermectin can have a positive impact on the healthcare system. However, the present results do not provide sufficient support for the hypothesis that ivermectin could be an alternative to COVID-19 vaccines.

Due to a large number of participants, this citywide program was unable to supervise whether ivermectin users were using ivermectin regularly, although the accumulated number of ivermectin tablets was strictly controlled. This occurred to be a potential conservative bias since the effects of ivermectin on prophylaxis could be underestimated due to adherence to the recommended frequency of ivermectin use.

While ivermectin is a multi-target drug [36], its maximum benefits occur when it is present at a minimum concentration in a wide range of sites to inhibit multiple metabolic and inflammatory pathways. However, although the dose of ivermectin employed in the program was smaller than the minimum to reach the concentration required to act in these multiple sites, the reduction in infection, mortality, and death rates in the infected group that used ivermectin prophylactically was surprisingly lower. Long-term or accumulated ivermectin could also play a critical role in its long-term protection against COVID-19.

Limitations

Being a prospective observational study that allowed subjects to self-select between treatment vs. non-treatment instead of relying on randomization, important confounders may have been differentially present, which could otherwise explain the differences observed. Given that the benefits measured occurred despite negative risk factors being more present in the treatment group, this suggests the benefits are likely accurate and unbiased. Further, studies relying on PSM techniques have been shown to consistently agree with those employing randomization [37,38], again supporting the likelihood that the benefits measured are accurate. The prevailing type of SARS-CoV-2 in the city was unknown due to the lack of genotyping surveillance during the period of the program. Whether the prophylaxis proposed in this program would be as effective in other SARS-CoV-2 variants is unclear. Also, there was no strict control on whether infected subjects used any specific drug in case of COVID-19 infection, and this allows the possibility that the differences may be explained by differences in the use of ivermectin or other medications as treatment.

Final discussion

In this citywide ivermectin prophylaxis program, a large, statistically significant decrease in mortality rate was observed after the program began among the entire population of city residents. When comparing subjects that used ivermectin regularly, non-users were two times more likely to die from COVID-19 while ivermectin users were 7% less likely to be infected with SARS-CoV-2 (p = 0.003).

Although this study is not a randomized, double-blind, placebo-controlled clinical trial, the data were prospectively collected and resulted in a massive study sample that allowed adjustment for numerous confounding factors, thus strengthening the findings of the present study.

Due to the well-established, long-term safety profile of ivermectin, with rare adverse effects, the absence of proven therapeutic options to prevent death caused by COVID-19, and lack of effectiveness of vaccines in real-life all-cause mortality analyses to date, we recommend that ivermectin be considered as a preventive strategy, in particular for those at a higher risk of complications from COVID-19 or at higher risk of contracting the illness, not as a substitute for COVID-19 vaccines, but as an additional tool, particularly during periods of high transmission rates.

## Conclusions

In a citywide ivermectin program with prophylactic, optional ivermectin use for COVID-19, ivermectin was associated with significantly reduced COVID-19 infection, hospitalization, and death rates from COVID-19.

## Figures and Tables

**Figure 3:**
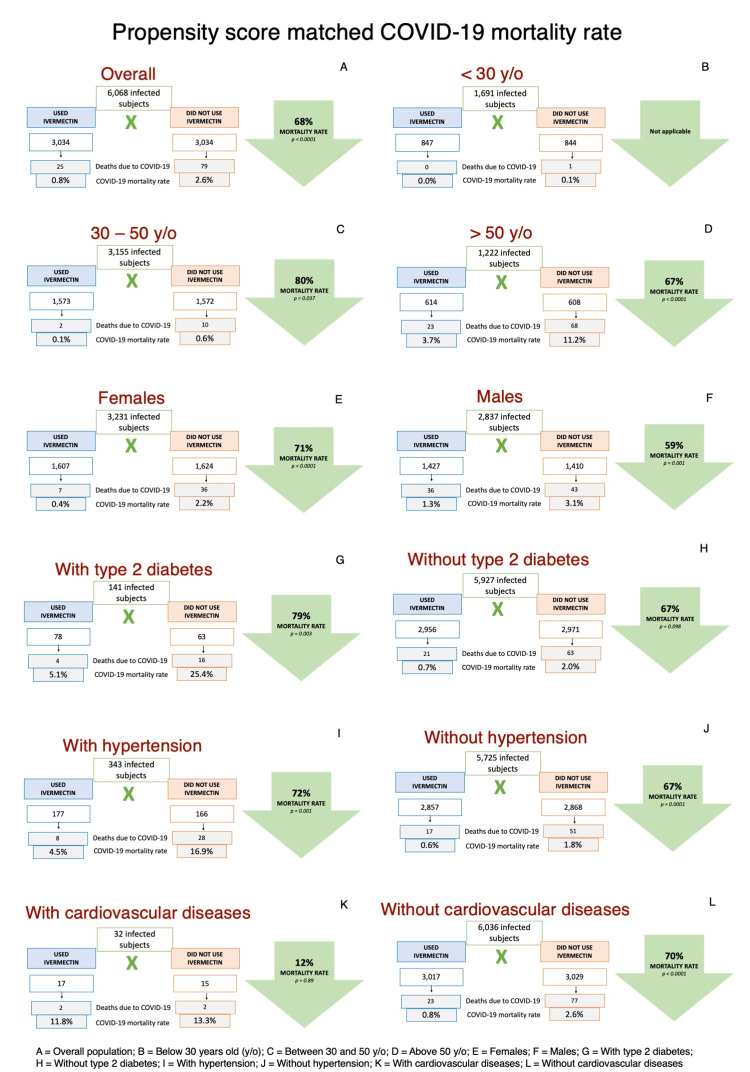
Propensity score-matched COVID-19 mortality rates in subpopulations.

**Figure 4:**
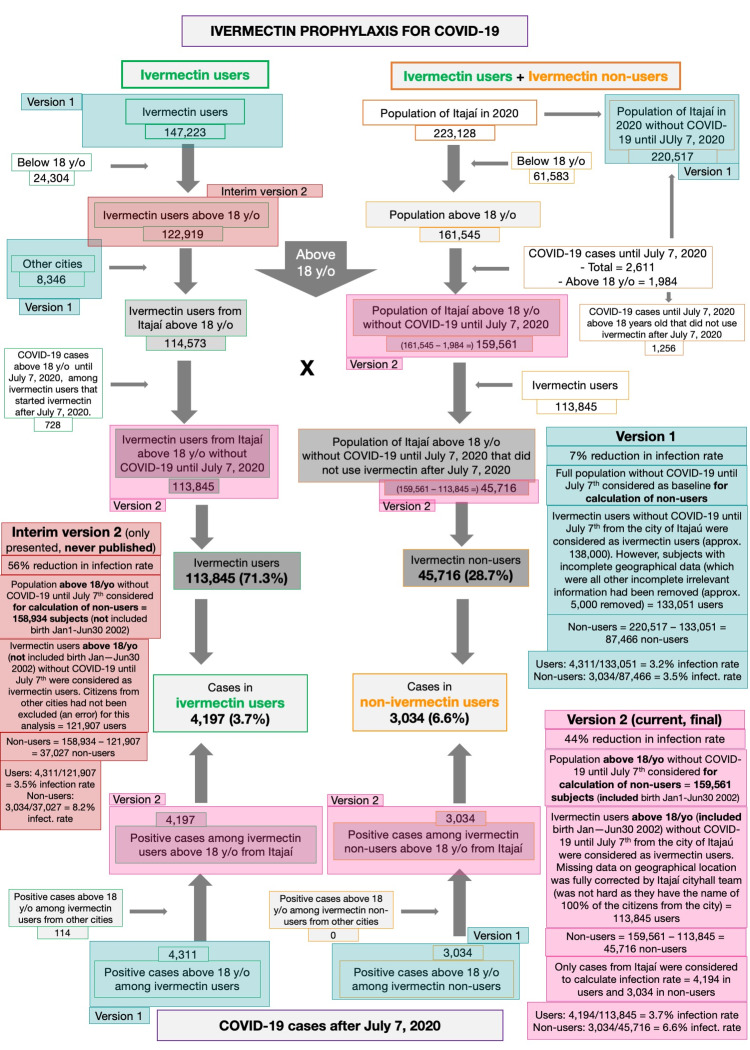
Modifications in the reported reduction in infection rate with ivermectin prophylaxis for COVID-19.

## Appendices

Table of contents

STROBE checklist………………………………………………………………………………

Unmatched analysis of infected patients…………………….………………………………

Determinants of COVID-19 mortality before matching……………………………………

Ivermectin versus non-ivermectin users in subpopulations………………….……………

Unmatched analysis……………………………………………………………………………

Propensity score-matched analysis…………………………………………….……………

Protocol modification for the calculation of infection rates…………………………….

STROBE checklist

Table 5 describes the STROBE (Strengthening the Reporting of Observational Studies in Epidemiology) checklist of this study.

**Table 5:** STROBE checklist. STROBE = Strengthening the Reporting of Observational Studies in Epidemiology.

Section	Item No.	Recommendation
Title and abstract	1	(a) Indicate the study’s design with a commonly used term in the title or the abstract - PRESENT IN BOTH TITLE (lines 2-3) AND ABSTRACT (lines 50-52)
		(b) Provide in the abstract an informative and balanced summary of what was done and what was found - BALANCED SUMMARY OF METHODS (lines 52-64) AND FINDINGS (lines 65-78)
Introduction		
Background/rationale	2	Explain the scientific background and rationale for the investigation being reported - SCIENTIFIC BACKGROUND (lines 111-165) AND RATIONALE (lines 167-173)
Objectives	3	State-specific objectives, including any prespecified hypotheses (lines 175-178)
Methods		
Study design	4	Present key elements of study design early in the paper (lines 185-190)
Setting	5	Describe the setting, locations, and relevant dates, including periods of recruitment, exposure, follow-up, and data collection (lines 190-235)
Participants	6	(a) Cohort study: Give the eligibility criteria and the sources and methods of selection of participants. Describe methods of follow-up (lines 237-275)
		(b) Cohort study: For matched studies, give matching criteria and number of exposed and unexposed (lines 177-288)
Variables	7	Clearly define all outcomes, exposures, predictors, potential confounders, and effect modifiers. Give diagnostic criteria, if applicable (lines 228-235; 277-281)
Data sources/measurement	8	For each variable of interest, give sources of data and details of methods of assessment (measurement). Describe comparability of assessment methods if there is more than one group (lines 277-311)
Bias	9	Describe any efforts to address potential sources of bias (lines 266-270; 313-317)
Study size	10	Explain how the study size was arrived at (lines 261-264)
Quantitative variables	11	Explain how quantitative variables were handled in the analyses. If applicable, describe which groupings were chosen and why (lines 293-311)
Statistical methods	12	(a) Describe all statistical methods, including those used to control for confounding (lines 293-320)
		(b) Describe any methods used to examine subgroups and interactions (lines 301-311)
		(c) Explain how missing data were addressed 313-317
		(d) Cohort study: If applicable, explain how the loss to follow-up was addressed - NO LOSS OF FOLLOW-UP
		(e) Describe any sensitivity analyses (lines 301-303; 310-311)
Results		
Participants	13	(a) Report numbers of individuals at each stage of the study, e.g., numbers potentially eligible, examined for eligibility, confirmed eligible, included in the study, completing follow-up, and analyzed (lines 331-338)
		(b) Give reasons for non-participation at each stage - NOT APPLICABLE
		(c) Consider the use of a flow diagram - NOT APPLICABLE
Descriptive data	14	(a) Give characteristics of study participants (e.g., demographic, clinical, and social) and information on exposures and potential confounders (lines 342-347 and Table 1)
		(b) Indicate the number of participants with missing data for each variable of interest - NO MISSING DATA
		(c) Cohort study: Summarize follow-up time (e.g., average and total amount) (lines 266-267)
Outcome data	15	Cohort study: Report numbers of outcome events or summary measures over time (lines 336-338; 357-359; 364-365; 390-395; Tables 2-3 and Figure 1)
Main results	16	(a) Give unadjusted estimates and, if applicable, confounder-adjusted estimates and their precision (e.g., 95% confidence interval). Make clear which confounders were adjusted for and why they were included (lines 338-340; 359-362; 365-367; 379-389; 394-398, Tables 2-4 and Figure 1)
		(b) Report category boundaries when continuous variables were categorized - NOT APPLICABLE
		(c) If relevant, consider translating estimates of relative risk into absolute risk for a meaningful time period - NOT APPLICABLE
Other analyses	17	Report other analyses done, e.g., analyses of subgroups and interactions, and sensitivity analyses (APPENDIX – pages 3- 8)
Discussion		
Key results	18	Summarize key results with reference to study objectives (lines 435-438)
Limitations	19	Discuss limitations of the study, taking into account sources of potential bias or imprecision. Discuss both direction and magnitude of any potential bias (lines 522-535)
Interpretation	20	Give a cautious overall interpretation of results considering objectives, limitations, the multiplicity of analyses, results from similar studies, and other relevant evidence (lines 440-518)
Generalizability	21	Discuss the generalizability (external validity) of the study results (lines 564-569)
Other information		
Funding	22	Give the source of funding and the role of the funders for the present study and, if applicable, for the original study on which the present article is based (lines 600-602)

Unmatched analysis of infected patients

Table 6 compares the hospitalization and mortality rates from COVID-19 infected patients between ivermectin users and non-users. Of the 7,345 subjects with COVID-19, there were 185 hospitalizations (2.52% hospitalization rate) among the non-users. Of the 4,311 ivermectin users, there were 86 hospitalizations (2.0% hospitalization rate), while among the 3,034 ivermectin non-users, there were 99 hospitalizations (3.3% hospitalization rate), with a reduction in hospitalization rate due to COVID-19 of 39% (RR, 0.61; 95% CI, 0.46-0.81; p = 0.0007). After adjustment for variables, reduction in hospitalization rate was 59% (RR < 0.41; 95% CI, 0.31-0.55; p < 0.0001).

**Table 6:** Pre-matching infection, hospitalization, death, and mortality rate among ivermectin users and non-users. IVM = ivermectin; CI = confidence interval. * Only subjects hospitalized in public hospitals. ** All deaths, including from public and private hospitals, and in-home.

		Overall	Ivermectin users	Non-IVM users	Risk ratio (95% CI) and p-value [p]	Adjusted risk ratio (95% CI) and p-value [p]
	Overall population (n)	159,561	113,845 (71.3%)	45,716 (28.7%)	-	-
COVID-19 infection	Infected population in the city of Itajaí (n)	7,345	4,197	3,034	-	-
	Infection rate (%)	4.6%	3.7%	6.6%	0.56 (0.53-0.58) [<0.0001]	-
	Infected population considered for the analysis (n)	7,345	4,311	3,034		
COVID-19 hospitalization	Hospitalization due to COVID-19*	185	86	99	-	-
	Hospitalization rate (in case of COVID-19) (%)	2.5%	2.0%	3.3%	0.61 (0.46-0.81) [0.0007]	0.41 (0.31-0.55) [<0.0001]
COVID-19 death	COVID-19 deaths (n)	141	62	79	-	-
	Risk of dying from COVID-19 in Itajaí (%)	0.09%	0.054%	0.173%	0.31 (0.23-0.44) [<0.0001]	-
	Mortality rate (among infected subjects) (%)	1.9%	1.4%	2.6%	0.55 (0.40-0.77) [0.0004]	0.43 (0.32-0.59) [<0.0001]

Among the 7,345 subjects from both groups with COVID-19, there were 141 deaths (1.9% mortality rate). Among the 4,311 ivermectin users, there were 62 deaths (1.4% mortality rate), while among the 3,034 subjects who did not use ivermectin prophylactically, there were 79 deaths (2.6% mortality rate), with a reduction in mortality rate of 45% (RR, 0.55; 95% CI, 0.40-0.77; p = 0.0004). When adjusted for residual variables, reduction in COVID-19 mortality rate was 57% (RR, 0.43; 95% CI, 0.32-0.59; p < 0.0001).

Determinants of COVID-19 mortality before matching

Table 7 describes the risk factors associated with death amongst the overall population before PSM. In unmatched analysis, unadjusted risk factors for COVID-19 among all participants included ivermectin non-users (p = 0.0004), age (p < 0.0001), sex (p = 0.014), T2D (p < 0.0001), hypertension (p < 0.0001), asthma (p = 0.041), COPD (p < 0.0001), cancer (overall) (p = 0.004), CVD (p < 0.0001), pulmonary diseases other than asthma and COPD (p = 0.003), and history of stroke (p < 0.0001). After adjustment for variables, ivermectin non-users (p < 0.0001), age (p < 0.0001), sex (p = 0.002), race (p = 0.052), T2D (p = 0.008), and pulmonary diseases other than asthma and COPD (p = 0.024) were demonstrated to be risk factors.

**Table 7:** Pre-matching COVID-19 mortality rate according to each characteristic in the overall population, ivermectin users, and non-users. COPD = chronic obstructive pulmonary disease; MI = myocardial infarction.

	Pre-matching			
Variable	Overall (n = 7,345)	Death (%)	Unadjusted COVID-19 mortality risk ratio and p-value [p]	Multivariate adjusted p-value
Ivermectin use - n (%)			0.55 (0.40-0.77) [0.0004]	<0.0001
Yes	4,311	62 (1.4%)		
No	3,034	79 (2.6%)		
Age - n (%)			[<0.0001]	<0.0001
<30 years old	2,336	0 (0.0%)		
30-50 years old	4,915	22 (0.45%)		
>50 years old	2,705	170 (6.28%)		
Sex - n (%)			0.66 (0.48-0.92) [0.014]	0.002
Female	3,983	62 (1.6%)		
Male	3,362	79 (2.4%)		
Race - n (%)			[0.20]	0.052
Caucasians	5,437	110 (2.0%)		
Afro-Brazilians	209	7 (3.3%)		
Mixed	1,583	22 (1.4%)		
Asian-Brazilians	114	2 (1.7%)		
Type 2 diabetes - n (%)			5.38 (3.59-8.06) [<0.0001]	0.008
Yes	214	27 (12.6%)		
No	7131	114 (1.6%)		
Hypertension - n (%)			6.57 (4.91-8.81) [<0.0001]	0.79
Yes	528	47 (8.9%)		
No	6,817	94 (1.4%)		
Asthma - n (%)			4.05 (1.06-15.5) [0.041]	0.27
Yes	26	2 (7.7%)		
No	7,319	139 (1.9%)		
COPD - n (%)			12.3 (4.48-33.5) [<0.0001]	0.11
Yes	13	3 (23.1%)		
No	7,332	138 (1.9%)		
Cardiovascular diseases - n (%)			6.46 (4.60-9.06) [<0.0001]	0.52
Yes	56	5 (8.9%)		
No	7,289	136 (1.9%)		
Other pulmonary diseases - n (%)			7.03 (1.91-25.8) [0.003]	0.024
Yes	15	2 (13.3%)		
No	7,330	139 (1.9%)		
Cancer (any type) - n (%)			4.97 (1.67-14.8) [0.004]	0.65
Yes	32	3 (9.4%)		
No	7,313	138 (1.9%)		
Current smoking - n (%)			1.43 (0.46-4.42) [0.53]	0.74
Yes	110	3 (2.7%)		
No	7,235	138 (1.9%)		
History of MI - n (%)			3.49 (0.52-23.4) [0.20]	0.91
Yes	15	1 (6.7%)		
No	7,330	140 (1.9%)		
History of stroke - n (%)			15.5 (6.58-27.1) [<0.0001]	0.13
Yes	21	6 (28.6%)		
No	7,324	135 (1.8%)		

Ivermectin versus non-ivermectin users in subpopulations

Tables 8, 9 depict the differences in mortality rate in different subpopulations of ivermectin users and ivermectin non-users, and compare mortality rates in each subpopulation between ivermectin users and non-users, before and after matching, respectively.

**Table 8:** Pre-matching COVID-19 mortality rate according to each characteristic in ivermectin users and ivermectin non-users, and mortality rate between ivermectin users versus non-users in each group. CI = confidence interval; n/a = not applicable; COPD = chronic obstructive pulmonary disease; MI = myocardial infarction.

	Ivermectin users				Non-ivermectin users				Users versus non-users
Variable	N (n = 4,311)	Mortality rate among ivermectin users (%)	Unadjusted COVID-19 mortality risk ratio (95% CI) and p-value [p]	Multivariate adjusted p-value	N (n = 3,034)	Mortality rate among non-ivermectin users (%)	Unadjusted COVID-19 mortality risk ratio (95% CI) and p-value [p]	Multivariate adjusted p-value	COVID-19 mortality risk ratio comparing ivermectin users versus non-users (95% CI) [p-value]
Age			[<0.0001]	<0.0001			[<0.0001]	<0.0001	
<30 years old	886	0 (0.0%)			844	1 (0.1%)			0.32 (0.01-7.78) [0.48]
31-49 years old	2,119	2 (0.1%)			1,572	10 (0.6%)			0.15 (0.03-0.68) [0.014]
>50 years old	1,304	60 (4.6%)			608	68 (11.2%)			0.41 (0.30-0.57) [<0.0001]
Sex			[0.044]	0.14			[0.15]	0.012	
Female	2,359	26 (1.1%)			1,624	36 (2.2%)			0.50 (0.30-0.82) [0.006]
Male	1,952	36 (1.8%)			1,410	43 (3.1%)			0.60 (0.39-0.94) [0.024]
Race			0.55	0.079			-	0.74	
Caucasians	3,245	48 (1.5%)			2,192	62 (2.8%)			0.52 (0.36-0.76) [0.0007]
Afro-Brazilians	109	3 (2.7%)			100	4 (4.0%)			0.69 (0.16-3.00) [0.62]
Mixed	901	10 (1.1%)			682	12 (1.8%)			0.63 (0.27-1.45) [0.28]
Asian-Brazilians	56	1 (1.8%)			60	1 (1.7%)			1.07 (0.07-16.7) [0.96]
Type 2 diabetes			5.94 (3.16-11.2) [<0.0001]	0.089			12.0 (7.35-19.5) [<0.0001]	0.024	
Yes	151	11 (7.3%)			63	16 (25.4%)			0.29 (0.14-0.58) [0.0006]
No	4,160	51 (1.2%)			2,971	63 (2.0%)			0.58 (0.40-0.83) [0.003]
Hypertension			4.82 (2.84-8.18) [<0.0001]	0.97			8.95 (5.79-13.8) [<0.0001]	0.29	
Yes	362	19 (5.2%)			166	28 (16.9%)			0.33 (0.19-0.57) [0.0001]
No	3,949	43 (1.1%)			2,868	51 (1.8%)			0.61 (0.40-0.91) [0.017]
Cardiovascular diseases			5.30 (1.73-16.2) [0.003]	0.40			5.40 (1.46-20.0) [0.012]	0.87	
Yes	41	3 (7.3%)			15	2 (13.3%)			0.55 (0.10-2.97) [0.49]
No	4,270	59 (1.4%)			3,019	77 (2.6%)			0.56 (0.40-0.78) [0.0007]
Asthma			3.52 (0.51-24.1) [0.20]	0.34			6.47 (1.07-39.2) [0.042]	0.59	
Yes	20	1 (5.0%)			6	1 (16.7%)			0.30 (0.02-4.11) [0.90]
No	4,291	61 (1.4%)			3,028	78 (2.6%)			0.55 (0.40-0.77) [0.0004]
COPD			20.5 (6.19-67.9) [<0.0001]	0.068			6.47 (1.07-39.2) [0.042]	0.69	
Yes	7	2 (28.6%)			6	1 (16.7%)			1.71 (0.20-14.5) [0.62]
No	4,304	60 (1.4%)			3,028	78 (2.6%)			0.54 (0.39-0.75) [0.0003]
Other pulmonary diseases			7.05 (1.08-46.0) [0.041]	0.26			9.70 (1.75-53.7) [0.009]	0.16	
Yes	10	1 (10.0%)			4	1 (20.0%)			0.40 (0.03-4.96) [0.48]
No	4,301	61 (1.4%)			3,029	78 (2.6%)			0.55 (0.39-0.77) [0.0004]
Cancer (any type)			7.20 (1.89-27.5) [0.004]	0.62			3.23 (0.49-21.4) [0.22]	0.96	
Yes	20	2 (10.0%)			12	1 (8.3%)			1.20 (0.12-11.9) [0.88]
No	4,291	60 (1.4%)			3,022	78 (2.6%)			0.54 (0.39-0.76) [0.0003]
Current smoking			2.25 (0.56-8.99) [0.25]	0.51			0.81 (0.12-5.73) [0.84]	0.58	
Yes	63	2 (3.2%)			47	1 (2.1%)			1.49 (0.14-16.0) [0.74]
No	4,248	60 (1.4%)			2,987	78 (2.6%)			0.54 (0.39-0.75) [0.0003]
History of MI			2.87 (0.19-43.8) [0.44]	-			9.71 (1.75-53.8) [0.009]	0.49	
Yes	11	0 (0.0%)			4	1 (25.0%)			0.14 (0.01-2.87) [0.20]
No	4,300	62 (1.4%)			3,030	78 (2.6%)			0.56 (0.40-0.78) [0.0006]
History of stroke			13.0 (3.63-46.8) [0.0001]	0.72			16.1 (7.31-35.6) [<0.0001]	0.15	
Yes	11	2 (18.2%)			10	4 (40.0%)			0.45 (0.11-1.97) [0.29]
No	4,300	60 (1.4%)			3,024	75 (2.5%)			0.56 (0.40-0.79) [0.0008]

**Table 9:** Propensity score-matched COVID-19 mortality rate according to each characteristic in ivermectin users and ivermectin non-users, and mortality rate between ivermectin users versus non-users in each group. PSM = propensity score matching; CI = confidence interval; n/a = not applicable; COPD = chronic obstructive pulmonary disease; MI = myocardial infarction.

	Ivermectin users				Non-ivermectin users				Users versus non-users
Variable	N (n = 3,034)	Death (%)	Unadjusted COVID-19 mortality risk ratio (95% CI) and p-value [p]	Multivariate adjusted p-value	N (n = 3,034)	Death (%)	Unadjusted COVID-19 mortality risk ratio (95% CI) and p-value [p]	Multivariate adjusted p-value	COVID-19 mortality risk ratio comparing Ivermectin users versus non-users [p-value]
Age			[<0.0001]	<0.0001			[<0.0001]	<0.0001	
<30 years old	847	0 (0.0%)			844	1 (0.1%)			n/a
30-50 years old	1,573	2 (0.1%)			1,572	10 (0.6%)			0.20 (0.04-0.91) [0.037]
>50 years old	614	23 (3.7%)			608	68 (11.2%)			0.33 (0.21-0.53) [<0.0001]
Sex			0.35 (0.14-0.82) [0.017]	0.014			0.73 (0.47-1.12) [0.15]	0.012	
Female	1,607	7 (0.4%)			1,624	36 (2.2%)			0.29 (0.18-0.46) [<0.0001]
Male	1,427	18 (1.3%)			1,410	43 (3.1%)			0.41 (0.24-0.71) [0.001]
Race			[0.33]	0.077			[0.74]	0.74	
Caucasians	2,206	17 (0.8%)			2,192	62 (2.8%)			0.28 (0.16-0.46) [<0.0001]
Afro-Brazilians	93	2 (2.1%)			100	4 (4.0%)			0.54 (0.10-2.87) [0.47]
Mixed	682	5 (0.7%)			682	12 (1.8%)			0.42 (0.15-1.18) [0.098]
Asian-Brazilians	53	1 (1.9%)			60	1 (1.7%)			1.13 (0.07-17.7) [0.93]
Type 2 diabetes	-		7.22 (2.54-20.5) [0.0002]	0.64			12.0 (7.35-19.5) [<0.0001]	0.24	
Yes	78	4 (5.1%)			63	16 (25.4%)			0.21 (0.07-0.59) [0.003]
No	2,956	21 (0.7%)			2,971	63 (2.1%)			0.33 (0.20-0.55) [0.098]
Hypertension			7.60 (3.32-17.4) [<0.0001]	0.99			8.95 (5.79-13.8) [<0.0001]	0.29	
Yes	177	8 (4.5%)			166	28 (16.9%)			0.28 (0.13-0.61) [0.001]
No	2,857	17 (0.6%)			2,868	51 (1.8%)			0.33 (0.19-0.58) [0.0001]
Cardiovascular diseases			15.4 (3.94-60.4) [0.0001]	0.90			5.40 (1.46-20.0) [0.012]	0.87	-
Yes	17	2 (11.8%)	-		15	2 (13.3%)			0.88 (0.14-5.52) [0.89]
No	3,017	23 (0.8%)			3,019	77 (2.6%)			0.30 (0.19-0.47) [<0.0001]
Asthma			8.99 (1.30-61.9) [0.026]	0.029			6.47 (1.07-39.2) [0.042]	0.59	
Yes	14	1 (6.7%)			6	1 (16.7%)			0.43 (0.03-5.78) [0.64]
No	3,019	24 (0.8%)			3,028	78 (2.6%)			0.31 (0.20-0.49) [<0.0001]
COPD	-		43.9 (13.2-146.1) [0.0001]	0.042			6.47 (1.07-39.2) [0.042]	0.70	
Yes	6	2 (33.3%)			6	1 (16.7%)			2.00 (0.24-16.6) [0.52]
No	3,028	23 (0.8%)			3,028	78 (2.6%)			0.30 (0.19-0.47) [<0.0001]
Other pulmonary diseases			n/a	0.89			9.70 (1.75-53.7) [0.009]	0.16	
Yes	4	0 (0.0%)			4	1 (20.0%)			n/a
No	3,030	25 (0.8%)			3,029	78 (2.6%)			0.30 (0.19-0.47) [<0.0001]
Cancer (any type)			n/a	0.85			3.23 (0.49-21.4) [0.22]	0.96	
Yes	10	0 (0.0%)			12	1 (8.3%)			n/a
No	3,240	25 (0.8%)			3,022	78 (2.6%)			0.32 (0.20-0.50) [<0.0001]
Current smoking			2.59 (0.36-18.8) [0.35]	0.68			0.81 (0.12-5.73) [0.84]	0.57	
Yes	48	1 (2.1%)			47	1 (2.1%)			0.97 (0.06-15.2) [0.99]
No	2,986	24 (0.8%)			2,987	78 (2.6%)			0.31 (0.20-0.48) [<0.0001]
History of MI			n/a	0.91			9.71 (1.75-53.8) [0.009]	0.49	
Yes	4	0 (0.0%)			4	1 (25.0%)			n/a
No	3,030	25 (0.8%)			3,030	78 (2.6%)			0.32 (0.20-0.50) [<0.0001]
History of stroke			23.9 (6.40-89.3) [<0.0001]	0.90			16.1 (7.31-35.6) [<0.0001]	0.15	
Yes	11	2 (18.2%)			10	4 (40.0%)			0.45 (0.10-1.97) [0.29]
No	3,023	23 (0.8%)			3,024	75 (2.5%)			0.32 (0.20-0.50) [<0.0001]

Unmatched analysis

Before matching (Table 8), unadjusted values showed that risk factors for both ivermectin users and non-users were aging (p < 0.0001 for both), T2D (p < 0.0001 for both), hypertension (p < 0.0001 for both), CVD (p = 0.003 and p = 0.012, respectively), COPD (p < 0.0001 and p = 0.042, respectively), other pulmonary diseases (p = 0.041 and p = 0.009, respectively), and history of stroke (p = 0.0001 and p < 0.0001, respectively). Male sex and cancer were risk factors for ivermectin users (p = 0.044 and p = 0.22, respectively). History of MI was a risk factor for ivermectin non-users (p = 0.009).

After adjustment for variables, remaining independent risk factors include aging for both ivermectin users (p < 0.0001) and non-users (p < 0.0001), male sex for non-users (p = 0.012), and T2D for ivermectin non-users (p = 0.024).

Mortality rates between ivermectin users were statistically lower than non-users among the following groups: between 31 and 49 years old (RR, 0.15; 95% CI, 0.03-0.68; p = 0.014), above 50 years old (RR, 0.41; 95% CI, 0.30-0.57; p < 0.0001), male sex (RR, 0.60; 95% CI, 0.39-0.94; p = 0.024), female sex (RR, 0.50; 95% CI, 0.30-0.82; p = 0.006), Caucasians (RR, 0.52; 95% CI, 0.36-0.76; p = 0.0007), subjects with T2D (RR, 0.29; 95% CI, 0.14-0.58; p = 0.0006), with hypertension (RR, 0.33; 95% CI, 0.19-0.57; p = 0.0001), and subjects without hypertension, T2D, COPD, asthma, other pulmonary diseases, CVD, history of MI, history of stroke, and non-smokers (RR, 0.54-0.61; 95% CI, 0.19-0.91; p = 0.0003 to 0.017).

Relative reduction of mortality risk rate with ivermectin use was more substantial in those with major common comorbidities, including T2D (71% reduction among subjects with T2D versus 42% reduction among subjects without T2D), hypertension (67% reduction in the COVID-19 death rate among subjects with hypertension versus 39% reduction among subjects without hypertension), asthma (70% reduction in the COVID-19 death rate among subjects with asthma versus 45% among subjects without asthma), and history of MI (86% reduction in the COVID-19 death rate among subjects with a history of MI versus 44% among subjects without a history of MI). Reduction of death risk was higher in females (50%) than in males (40%), in Caucasians (48%) than in mixed-race subjects (37%) and afro-Brazilians (31%), and between 30 and 50 years old (85%) than above 50 years old (59%). However, the absolute risk reduction was higher among those above 50 years old (6.6 points percent [p.p.]) than those between 30 and 50 years old (0.5 p.p.) and below 30 years old (0.1 p.p.).

Propensity score-matched analysis

Table 9 describes propensity score-matched mortality rates in subpopulations of ivermectin users and ivermectin non-users and then compares ivermectin users and non-users for each characteristic. Figure 3 illustrates COVID-19 mortality rates in subpopulations after matching. Post-matching mortality rates, risk ratios, and p-values among ivermectin non-users remained the same as before matching. Among ivermectin users, the values were as follows: aging (p < 0.0001), male sex (p = 0.017), T2D (p = 0.0002), hypertension (p < 0.0001), CVD (p = 0.0001), asthma (p = 0.026), COPD (p = 0.0001), and history of stroke (p < 0.0001). There were no deaths in ivermectin users with other pulmonary diseases, cancer, and a history of MI.

After PSM, the ratio between mortality rates of ivermectin users and ivermectin non-users showed statistical reduction in mortality rate with ivermectin use in subjects above 30 years old (30-50 years old; RR, 0.20; 95% CI, 0.04-0.91; p = 0.037; >50 years old; RR, 0.33; 95% CI, 0.21-0.53; p < 0.0001), in both sexes (male sex; RR, 0.41; 95% CI, 0.24-0.71; p = 0.001; female sex; RR, 0.29; 95% CI, 0.18-0.46; p < 0.0001), Caucasians (RR, 0.28; 95% CI, 0.16-0.46; p < 0.0001), subjects with T2D (RR, 0.21; 95% CI, 0.07-0.59; p = 0.003), with hypertension (RR, 0.28; 95% CI, 0.13-0.61; p = 0.001), and subjects without hypertension, T2D, COPD, asthma, other pulmonary diseases, CVD, history of MI, history of stroke, and non-smokers (RR, 0.30-0.32; 95% CI, 0.19-0.58; p < 0.0001 for all, except for no diabetes, p = 0.098).

After matching, relative reductions in mortality risk with the use of ivermectin was slightly higher in subjects with T2D (79% and 67% reduction among subjects with T2D and without T2D, respectively) and hypertension (72% and 67% reduction in COVID-19 mortality rate in subjects with and without hypertension, respectively), but not with other comorbidities. The absolute risk reduction was higher among those above 50 years old, of 75 subjects saved for every 1,000 subjects infected with COVID-19 (7.5 p.p.) than those between 30 and 50 years old (0.5 p.p.; five subjects saved for every 1,000 COVID-19 cases) and below 30 years old (0.1 p.p.; one subject saved for every 1,000 COVID-19 cases).

Protocol modification for the calculation of infection rates

Previously, we had considered the full population of Itajaí as the source for the calculation of ivermectin non-users, which falsely raised the number of non-users and, consequently, falsely reduced the infection rate among ivermectin non-users. We also excluded subjects below 18 years old and participating subjects from other cities, since their outcomes would not be accounted for in the statistics of the city of Itajaí. Figure 4 summarizes the modifications.
