# Correction: Ivermectin Prophylaxis Used for COVID-19: A Citywide, Prospective, Observational Study of 223,128 Subjects Using Propensity Score Matching

**DOI:** 10.7759/cureus.c61

**Published:** 2022-03-24

**Authors:** Lucy Kerr, Flavio A Cadegiani, Fernando Baldi, Raysildo B Lobo, Washington Luiz O Assagra, Fernando Carlos Proença, Pierre Kory, Jennifer A Hibberd, Juan J Chamie-Quintero

**Affiliations:** 1 Medicine, Instituto Kerr, São Paulo, BRA; 2 Clinical Endocrinology, Corpometria Institute, Brasilia, BRA; 3 Clinical Endocrinology, Applied Biology Inc, Irvine, USA; 4 Animal Sciences, Universidade Estadual de São Paulo (UNESP), São Paulo, BRA; 5 Genetics, Universidade de São Paulo, Ribeirão Preto, BRA; 6 Genetics, Centro Técnico de Avaliação Genômica - C.T.A.G., Ribeirão Preto, BRA; 7 Bioinformatics, Itajaí City Hall, Itajaí, BRA; 8 Internal Medicine, Front Line COVID-19 Critical Care Alliance (FLCCC), Madison, USA; 9 Dentistry, University of Toronto, Toronto, CAN; 10 Data Analysis, Universidad EAFIT, Medellín, COL

It has come to the attention of the journal that several authors failed to disclose all relevant conflicts of interest when submitting this article. As a result, Cureus is issuing the following erratum and updating the relevant conflict of interest disclosures to ensure these conflicts of interest are properly described as recommended by the ICMJ:

- **Lucy Kerr:** Paid consultant for both Vitamedic, an ivermectin manufacturer, and Médicos Pela Vida (MPV), an organization that promotes ivermectin as a treatment for COVID-19.
- **Flavio A. Cadegiani:** Paid consultant ($1,600.00 USD) for Vitamedic, an ivermectin manufacturer. Dr. Cadegiani is a founding member of the Front Line COVID-19 Critical Care Alliance (FLCCC), an organization that promotes ivermectin as a treatment for COVID-19.
- **Pierre Kory: **President and Chief Medical Officer of the Front Line COVID-19 Critical Care Alliance (FLCCC), an organization that promotes ivermectin as a treatment for COVID-19. Dr. Kory reports receiving payments from FLCCC. In February of 2022, Dr. Kory opened a private telehealth fee-based service to evaluate and treat patients with acute COVID, long haul COVID, and post-vaccination syndromes.
- **Jennifer A. Hibberd:** Co-founder of the Canadian Covid Care Alliance and World Council for Health, both of which discourage vaccination and encourage ivermectin as a treatment for COVID-19.
- **Juan J. Chamie-Quintero:** Contributor to the Front Line COVID-19 Critical Care Alliance (FLCCC) and lists the FLCCC as his employer on his LinkedIn page.

